# The potential predictive value of tumor budding for neoadjuvant chemoradiotherapy response in locally advanced rectal cancer

**DOI:** 10.1007/s00066-018-1340-0

**Published:** 2018-08-01

**Authors:** Tarkan Jäger, Daniel Neureiter, Mohammad Fallaha, Philipp Schredl, Tobias Kiesslich, Romana Urbas, Eckhard Klieser, Josef Holzinger, Felix Sedlmayer, Klaus Emmanuel, Adam Dinnewitzer

**Affiliations:** 1 Department of Surgery, Paracelsus Medical University, Muellner Hauptstraße 48, 5020 Salzburg, Austria; 2 Institute of Pathology, Paracelsus Medical University, Muellner Hauptstraße 48, 5020 Salzburg, Austria; 3 Faculty of Medicine, Imperial College School of Medicine, South Kensington, SW7 2AZ London, UK; 4 Laboratory for Tumor Biology and Experimental Therapies (TREAT), Institute for Physiology and Pathophysiology, Salzburg, Austria; 5 Department of Internal Medicine, Paracelsus Medical University, Muellner Hauptstraße 48, 5020 Salzburg, Austria; 6 Department of Radiotherapy and Radiation Oncology, Paracelsus Medical University, Muellner Hauptstraße 48, 5020 Salzburg, Austria

**Keywords:** Neoadjuvant therapy, Tumor microenvironment, Epithelial–mesenchymal transition, Colorectal cancer, Prognostic factor, Neoadjuvante Therapie, Tumormikromilieu, Epithelial-mesenchymale Transition, Kolorektales Karzinom, Prognostische Faktoren

## Abstract

**Purpose:**

This study was conducted to investigate the potential predictive value of tumor budding for neoadjuvant chemoradiotherapy response in locally advanced rectal cancer.

**Patients and methods:**

Surgical specimens of 128 ypUICC (Union for International Cancer Control) stage 0–III mid-to-low rectal cancer patients were identified from a prospectively maintained colorectal cancer database and classified into two groups using the 10 high-power field average method: none/mild tumor budding (BD-0) and moderate/severe tumor budding (BD-1). Overall survival, relapse-free survival (RFS), and recurrence estimates were calculated using the Kaplan–Meier method and compared with the log-rank test. For RFS, a multivariable Cox’s proportional hazards regression analysis was performed.

**Results:**

No (*n* = 20) or mild (*n* = 27) tumor budding (BD-0) was identified in 47 (37%) and moderate (*n* = 52) or severe (*n* = 29) tumor budding (BD-1) in 81 (63%) surgical specimens. Positive tumor budding (BD-1) was associated with significantly reduced T‑level downstaging (*P* < 0.001) and tumor regression (*P* < 0.001). After a median follow-up time of 7 years (range 2.9–146.7 months), BD-0 patients had more favorable 5‑year RFS (90 vs. 71%, *P* = 0.02) and distant recurrence (2 vs. 12%, *P* = 0.03) estimates. Multivariable analyses confirmed BD-1 as a negative predictive parameter for RFS (hazard ratio = 3.44, 95% confidence interval 1.23–9.63, *P* = 0.018).

**Conclusions:**

Our data confirm tumor budding as a strong prognostic factor and its potential predictive value for neoadjuvant chemoradiotherapy response in locally advanced rectal cancer patients. This provides the opportunity to modify and individualize neoadjuvant therapy regimens for non-responders.

## Zusammenfassung

**Hintergrund und Ziele:**

Die Studie untersucht die Wertigkeit des „Tumor Budding“ als möglichen prädiktiven Faktor für ein Ansprechen auf die neoadjuvante Radiochemotherapie beim lokal fortgeschrittenen Rektumkarzinom.

**Patienten und Methoden:**

Resektionspräparate von 128 Patienten mit einem lokal fortgeschrittenen Rektumkarzinom im ypUICC (Union for International Cancer Control) Stadium 0–III wurden aus einer prospektiv angelegten Datenbank identifiziert und hinsichtlich „Tumor Budding“ in zwei Gruppen eingeteilt: BD-0 (kein bis mildes „Tumor Budding“) und BD-1 (mäßig bis hochgradiges „Tumor Budding“). Primäre Endpunkte der Studie waren die lokale Kontrolle, die Fernmetastasierung sowie das Gesamt- und das krankheitsfreie Überleben (RFS), die mit der Kaplan-Meier-Methode berechnet und mittels Log-rank-Test auf Signifikanz geprüft wurden. Für das RFS wurde mittels Cox-Regression multivariat getestet.

**Ergebnisse:**

Die retrospektive Analyse der Resektionspräparate ergab ein BD-0 bei 47 (37 %) und ein BD-1 bei 81 Patienten (63 %). Positives „Tumor Budding“ (BD-1) war signifikant mit einer erniedrigten Ansprechrate auf die neoadjuvante Radiochemotherapie sowohl hinsichtlich T‑Level-Downstaging (*P* < 0,001) als auch Tumorregression (*P* < 0,001) assoziiert. Dabei zeigten BD-0-Patienten ein signifikant besseres 5‑Jahres-RFS (90 vs. 71 %; *P* = 0,02) sowie eine bessere 5‑Jahres-Fernmetastasierungsrate (2 vs 12 %; *P* = 0,03). In der multivariablen Analyse wurde BD-1 als negativ prädiktiver Faktor für das RFS identifiziert (HR = 3,44; 95 %-Konfidenzintervall 1,23–9,63; *P* = 0,018).

**Schlussfolgerung:**

Unsere Daten bestätigen die Wertigkeit des „Tumor Budding“ als möglichen prädiktiven und prognostischen Faktor für ein Ansprechen auf die neoadjuvante Radiochemotherapie beim lokal fortgeschrittenen Rektumkarzinom. Damit könnte die Bestimmung des „Tumor Budding“ als Stratifikationsparameter für eine individualisierte neoadjuvante Therapie dienen.

## Introduction

The multidisciplinary approach including radiotherapy, chemotherapy, and surgery is regarded as the standard of care for locally advanced (T3/4 and/or node positive) mid-to-low rectal cancer patients [1, 2]. Neoadjuvant regimens consisting of preoperative long-course 5‑FU (5‑fluorouracil) based chemoradiotherapy (CRT; 45–50.4 Gy, 25–28 fractions) and preoperative short-course radiotherapy (25 Gy, five fractions) are considered the primary treatments of choice [3–5]. The CAO/ARO/AIO-94 trial demonstrated the superiority of preoperative CRT with respect to local control, treatment compliance, and overall toxicity profile, but not in overall survival benefit, when compared to postoperative CRT [2, 5]. However, in patients with a complete or near complete pathological response receiving preoperative CRT, there was an improvement in long-term outcomes independent of clinicopathologic parameters [6]. Further randomized controlled trials (SRCT, TME, FFCD9201, EORTC22912, TROG 01.04, and FOWARC) have confirmed the most beneficial impact of preoperative therapy (radiotherapy and/or chemotherapy) on local control [7–13].

Some aspects of preoperative therapy modalities such as the optimal radiotherapy fractionation, the interval between radiotherapy and surgery, or the inclusion of oxaliplatin are still under debate [13–21]. Out of seven randomized controlled studies evaluating the neoadjuvant use of oxaliplatin in the treatment of locally advanced rectal cancer, only two (FORWARC and CAO/ARO/AIO-04) demonstrated a beneficial effect on early endpoints (such as pathological complete response rate) [13, 19]. Furthermore, only the CAO/ARO/AIO-04 study demonstrated a significant improvement in disease-free survival [19].

Irrespective of a reliable tumor downstaging with 15–27% complete responders and stable local recurrence rates of 6%, more than half of the patients show no or just minor response to neoadjuvant therapy and develop distant recurrences in over 25% of cases [18, 22, 23]. Non-responsiveness exposes patients to the risks of toxicity whilst delaying surgery, for no apparent benefit [24]. Pretreatment identification of these patients and implementation of an individualized neoadjuvant therapy regimen may decrease recurrence rates and reduce perioperative complications.

While the classical tumor staging system (TNM) is accepted as the strongest predictor of clinical outcome, it has certain failings in stratifying patient subsets with intermediate tumor response after neoadjuvant therapy into more meaningful prognostic groups [25]. The assessment of tumor regression grading may overcome this shortcoming by measuring the degree of cellular tumor response to neoadjuvant therapy [6, 26, 27]. Both the TNM classification and tumor regression grading are only available in the resected specimen after neoadjuvant therapy and thus are not accessible in pretreatment specimens to assist with the planning of a modified neoadjuvant therapy.

Tumor budding is a promising histomorphological prognostic factor reported in 20–40% of colorectal cancer (CRC) cases [28]. It is defined as the presence of detached isolated single cancer cells or small clusters of up to four cells at the invasive front of epithelial cancers and is associated with lymphovascular invasion, distant metastases, and poor prognosis [28, 29]. Tumor buds in CRC specimens represent the histological phenotype of epithelial-to-mesenchymal transition (EMT) [30, 31]. This transition is characterized by a series of cell alterations (loss of cell adhesion molecules, cytoskeletal alterations, increased production of extracellular matrix components, resistance to apoptosis, and the ability to degrade basement membrane) resulting in a phenotype with increased migratory capacity and invasiveness [32–34]. An important factor in this process is that a subset of carcinoma cells acquire the properties of stem cells, promoting long-term tumor propagation, drug resistance, and development of metastases [35, 36].

Some challenges and caveats exist when interpreting the role of tumor budding in neoadjuvantly treated rectal cancer patients, as most of the existing knowledge is derived from colon and historical rectal cancer patients before the era of neoadjuvant therapy regimens. There is, however, a considerable lack of knowledge about the role of tumor budding in neoadjuvantly treated rectal cancer patients, with only six published articles to date in the English literature exploring this topic [24, 29, 37–40].

The aim of this retrospective cohort study was to examine the impact of tumor budding, analyzed in the resected specimens of 128 neoadjuvantly treated mid-to-low rectal cancer patients, on long-term outcome and to evaluate its potential predictive value for neoadjuvant chemoradiotherapy (nCRT) response.

## Materials and methods

### Patients

**Fig. 1:**
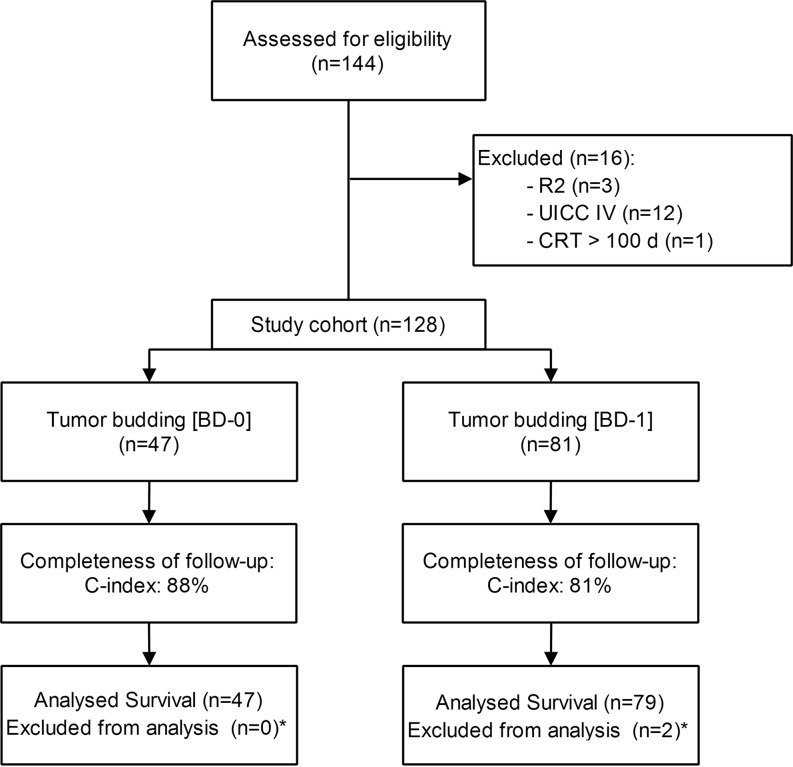
Flow chart of the study population. * death within 30 days after surgery, *CRT* chemoradiotherapy, *UICC* Union for International Cancer Control

The study cohort was identified from the institutional prospectively maintained colorectal cancer database including a well-characterized and previously published cohort of 144 patients [26]. All patients presented with locally advanced (cT3/4 and/or clinically node positive) mid-to-low rectal cancer who received nCRT prior to total mesorectal (TME) surgery after an interval of 4–6 weeks. Out of these 144 patients, 16 were excluded before analysis because of stage IV disease (*n* = 12), R2 resections (defined as incomplete local resection; *n* = 3), and an interval >90 days between end of nCRT and TME surgery (*n* = 1). The final study cohort comprised a total of 128 consecutive non-metastasized ypUICC (Union for International Cancer Control) stage 0–III rectal cancer patients treated with long-course nCRT and TME surgery between January 2003 and December 2012 at our tertiary care center (Fig. 1). Patients with R1 resection (defined as tumor present 1 mm or less from the radial/resection margin) remained in the study cohort. The resected specimens were assessed retrospectively with a standardized protocol for the presence of tumor budding (described below). Institutional review board approval was obtained to review records and report results.

### Neoadjuvant therapy

All patients received long-course nCRT, which consisted of either oral capecitabine or intravenously administered 5‑fluoruracil during 6 weeks of radiotherapy. One-hundred-eleven patients (87%) received a total dose of 45 Gy delivered in three or four high-energy photon beams by a three-dimensional conformal technique, in 25 fractions with daily fractional doses of 1.8 Gy (5 Fx/week) in 5 consecutive weeks. In ten patients (8%), a boost to the macroscopic tumor of up to 50.4 Gy was applied. The remaining six patients received a reduced total dose of 40 to 44 Gy. In approximately half of the patients (52%), oxaliplatin was used as an adjunct to the concomitant chemotherapy. Ten patients (8%) were treated within the ABCSG 95 (Austrian Breast & Colorectal Cancer Study Group) trial protocol (NCT00297141) [41, 42]. Failure to complete nCRT was an exclusion criterion.

### Pathologic examination and tumor budding

Pathology specimens were examined independently by three gastrointestinal pathologists (RU, EK, and DN) who were blinded to the patient’s outcome. All rectal specimens were processed macroscopically according to the national guidelines of the Austrian Society of Pathology with details published previously [43, 44]. Completeness of resection was scored as R0 for negative margins (regardless of distance between tumor and resection margins), R1 for microscopic tumor present 1 mm or less from the radial/resection margin, and R2 for gross residual tumor. Pathologic stage (ypT and ypN) was determined according to the 7^th^ AJCC TNM classification [25]. Additionally, the pathological response of the resected specimens to nCRT was graded according the four-category TRG system developed by the American Joint Committee on Cancer and the College of American Pathologists (AJCC/CAP), published elsewhere [26]: in short, complete response (AJCC/CAP grade 0) is characterized by no viable cancer cells, moderate response (AJCC/CAP grade 1) by single or small groups of cancer cells, minimal response (AJCC/CAP grade 2) by residual cancer outgrown by fibrosis, and finally poor response (AJCC/CAP grade 3) by minimal or no tumor cell death with extensive residual cancer in the resected specimen.

**Fig. 2:**
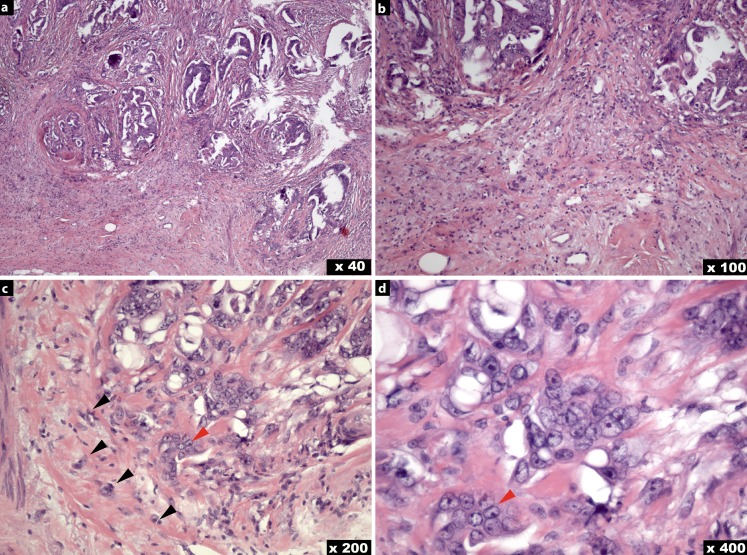
Illustration of a rectal adenocarcinoma with budding. Tumor buds, defined as individual cancer cells or small clusters of tumor cells at the invasive front (*arrows black/red* in **c**, **d**), are illustrated. H&E hematoxylin and eosin, original magnification **a** (×40), **b** (×100), **c** (×200), **d** (×400)

Budding focus was defined as the presence of isolated cancer cells or clusters of up to four cancer cells either within the tumor or at the invasive tumor front [45]. As proposed by Koelzer et al. [46], the 10 high-power fields (HPF) method was applied for a quantitative assessment of tumor budding: (i) All H&E slides with tumor areas of the resected specimen were continuously screened for the highest grade of tumor budding at low magnification (× 4 up to × 10). (ii) Based on this histomorphological preselection, tumor buds were then identified by the pathologist and counted in a total of 10 HPFs (× 40; Fig. 2a–d; [47]). Consecutively, the average number of buds in these 10 HPFs was calculated. Tumor budding was categorized as “none” if there was no budding focus (*n* = 20), as “mild” if there was up to one budding focus (*n* = 27), as “moderate” if there were more than one and less than five budding foci (*n* = 52), and as “severe” if more than five budding foci (*n* = 29) could be observed in the average of 10 HPF (400 × magnification). For further statistical analyses we then formed two groups: BD-0 (none or mild tumor budding; *n* = 47) and BD-1 (moderate or severe tumor budding; *n* = 81).

### Follow-up and oncologic outcomes

Patient follow-up was performed according to the guidelines of the Austrian Society of Surgical Oncology [48]. Outcome measures assessed in this study included survival estimates (overall [OS] and relapse-free survival [RFS]) and recurrence rates (overall [OR], distant [DR], and local recurrence [LR]). Time-to-event endpoints were calculated from the date of primary surgery. Overall survival was defined as time to death, irrespective of cause, and RFS as the time between surgery and the first recurrence event or death, irrespective of cause. Relapse of disease was defined as pathological, radiological, or clinical determination of rectal cancer recurrence confined to the prior pelvic treatment field (LR) or at any other site, including but not limited to the liver, lungs, and retroperitoneum (DR).

### Statistical analysis

Clinicopathological characteristics were compared between the two groups BD-0 and BD-1. Statistical significance in differences of clinicopathological variables were tested with the independent samples t‑test and Mann–Whitney U test for normally and non-normally distributed data, respectively. For the comparison of proportions, the χ^2^ test or Fisher exact test were used, as appropriate.

For the analyses of the 5‑year survival and recurrence estimates, all events after 60 months of follow-up and patients with a date of last contact more than 60 months after diagnosis were censored at 60 months. Loss to follow-up was assessed with the completeness of follow-up index C as described by Clark et al. [49]. This index quantifies the effect of losses to follow-up as the ratio of the total observed person-time of follow-up as a percentage of the potential time of follow-up. OS, RFS, and recurrence rates were estimated using the Kaplan–Meier method and compared with the log-rank test.

For RFS, a multivariable Cox proportional hazards model was calculated to adjust for baseline differences between BD-0 and BD-1 groups. All the predictors which had a *P*-value ≤0.10 in the univariate analyses were put in a forward step procedure into the model by keeping variables with a *P* < 0.05 and excluding those with a *P* > 0.05. All the tests are two-sided, and a *P*-value <0.05 indicates a statistically significant difference. Statistical analyses were performed with STATA release 14.2 (StataCorp LLC, College Station, TX).

## Results

### Patients

The clinical pretreatment characteristics and pathologic outcomes of the tumors in the BD-0 and BD-1 groups are shown in Tables 1 and 2. Between January 2003 and December 2012, 128 patients (87 males) with a mean age of 64 years (range 34–84 years) were identified for this retrospective cohort study. After a median interval of 5.1 weeks (range 2.7–9.3 weeks) from the end of nCRT, a low anterior (72%, 92 of 128) or an abdominoperineal rectal resection with TME (28%, 36 of 128) was performed. Six patients (5%) had positive circumferential resection margin involvement, defined as microscopic evidence of tumor cells 1 mm or less from the margin.

**Table 1:** Patient characteristics

	All (*n* = 128)		Tumor budding					
Characteristics			BD-0 (*n* = 47)		BD-1 (*n* = 81)		*P*-value	Test
*Age*							0.44	
Mean, years (SD)	64	(10)	63	(10)	64	(10)		T
*Gender, n (%)*							0.45	
Female	41	(32)	17	(41)	24	(59)		C
Male	87	(68)	30	(34)	57	(66)		
*ASA classification, n (%)*							0.44	
1	24	(19)	11	(46)	13	(54)		C
2	77	(60)	25	(32)	52	(68)		
3	27	(21)	11	(41)	16	(59)		
*BMI*							0.54	
Mean, kg/m^2^ (SD)	25.2	(3.9)	25.5	(4.1)	25.0	(3.8)		T
*Clinical UICC stage, n (%)*							0.60	
I	1	(1)	1	(100)	0	(0)		E
II	57	(45)	21	(37)	36	(63)		
III	66	(52)	23	(35)	43	(65)		
No. missing	4	(3)	2	–	2	–		
*Clinical T stage, n (%)*							0.008	
T1	–	–	–	–	–	–		E
T2	3	(2)	2	(67)	1	(33)		
T3	107	(84)	43	(40)	64	(60)		
T4	16	(13)	1	(6)	15	(94)		
No. missing	2	(2)	1	–	1	–		
*Clinical N stage, n (%)*							0.90	
N0	58	(45)	22	(38)	36	(62)		E
N1	56	(44)	20	(36)	36	(64)		
N2	10	(8)	3	(30)	7	(70)		
No. missing	4	(3)	2	–	2	–		
*Procedure, n (%)*							0.75	
APE	36	(28)	14	(39)	22	(61)		C
LAR	92	(72)	33	(36)	59	(64)		
*Other malignancies, n (%)*							0.07	
No	110	(86)	44	(40)	66	(60)		E
Yes	18	(14)	3	(17)	15	(83)		
*Time: end CRT to surgery*							0.68	
Median, weeks	5.1		5.1		5.1			M
(Range)	(2.7–9.3)		(2.9–8.9)		(2.7–9.3)			

*ASA* American Society of Anesthesiologists, *APE* abdominoperineal excision, *BD* tumor budding, *BMI* body mass index, *CRT* chemoradiotherapy, *LAR* low anterior resection, *UICC* Union for International Cancer Control, *SD* standard deviation

*C *χ^2^ test, *E* Fisher’s exact test, *T* Student t‑test, *M* Mann–Whitney U test

**Table 2:** Pathological characteristics

	All (*n* = 128)		Tumor Budding					
Characteristics			BD-0 (*n* = 47)		BD-1 (*n* = 81)		*P*-value	Test
*AJCC/CAP TRG, n (%)*							<0.001	
0	16	(13)	16	(100)	0	(0)		E
1	39	(30)	26	(67)	13	(33)		
2	54	(42)	3	(6)	51	(94)		
3	19	(15)	2	(11)	17	(89)		
*Histologic grade, n (%)*							0.15	
Well/moderate	106	(83)	42	(40)	64	(60)		E
Poor	22	(17)	5	(23)	17	(77)		
*ypUICC, n (%)*							<0.001	
0	15	(12)	15	(100)	0	(0)		E
I	38	(30)	22	(58)	16	(42)		
II	38	(30)	3	(8)	35	(92)		
III	37	(29)	7	(19)	30	(81)		
*Pathologic T stage, n (%)*							<0.001	
T0	16	(13)	16	(100)	0	(0)		E
T1	7	(5)	5	(71)	2	(29)		
T2	36	(28)	19	(53)	17	(47)		
T3	64	(50)	7	(11)	57	(89)		
T4	5	(4)	0	(0)	5	(100)		
*Pathologic N stage, n (%)*							0.01	
N−	92	(72)	40	(43)	52	(57)		C
N+	36	(28)	7	(19)	29	(81)		
*Nodes examined*							0.54	
Median (range)	14	(0–33)	14	(0–27)	14	(3–33)		M
Mean (SD)	13.6	(5.9)	13.1	(5.7)	14	(6.0)		
*Nodes involved*							0.02	
Median (range)	0	(0–14)	0	(0–12)	0	(0–14)		M
Mean (SD)	1	(2.4)	0.6	(2)	1.2	(2.5)		
*Lymphatic invasion, n (%)*							0.003	
No	90	(83)	40	(44)	50	(56)		E
Yes	19	(17)	2	(11)	17	(89)		
No. missing	19		5		14			
*Venous invasion, n (%)*							0.13	
No	105	(95)	42	(40)	63	(60)		E
Yes	6	(5)	0	(0)	6	(100)		
No. missing	17		5		12			
*Adjuvant Chemotherapy, n (%)*							0.70	
No	57	(45)	22	(39)	35	(61)		C
Yes	71	(55)	25	(35)	46	(65)		
*CRM, n (%)*							0.09	
>1 mm	121	(95)	46	(38)	75	(62)		E
≤1 mm	6	(5)	0	(0)	6	(100)		
No. missing	1		1		0			

*AJCC* American Joint Committee on Cancer, *BD* tumor budding, *CAP* College of American Pathologists, *CRM* circumferential resection margin, *TRG* tumor regression grading, *UICC* Union for International Cancer Control, *SD* standard deviation

*C* χ^2^ test, *E* Fisher’s exact test, *M* Mann–Whitney U test

**Fig. 3:**
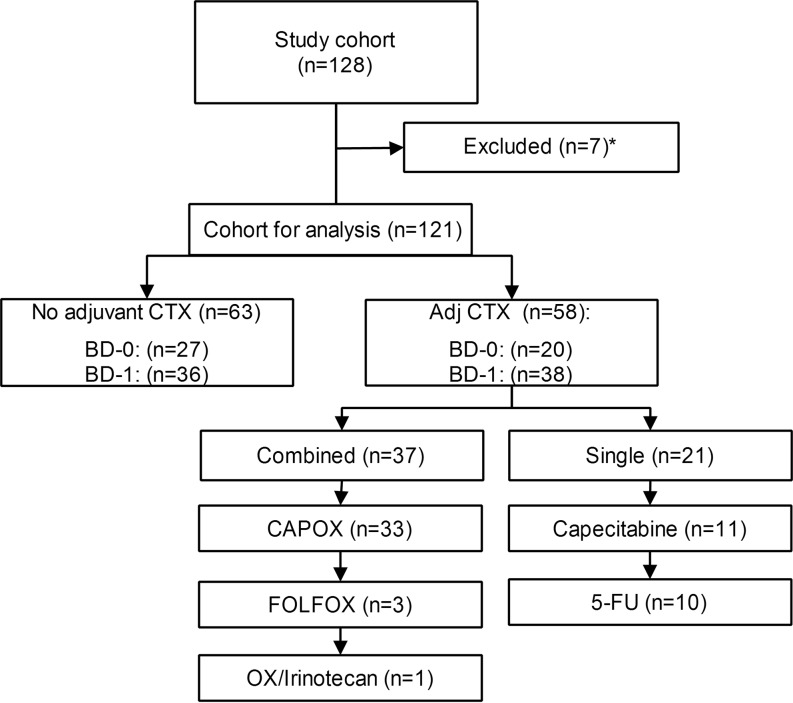
Flow chart of adjuvant chemotherapy in 128 neoadjuvant chemoradiotherapy treated locally advanced rectal cancer patients. * death within 30 days after surgery (*n* = 2), adjuvant chemotherapy status not available (*n* = 5), *Adj* adjuvant, *BD* tumor budding, *CTX* chemotherapy, *CAPOX* capecitabine combined with oxaliplatin, *FOLFOX* folinic acid combined with 5‑fluorouracil and oxaliplatin, *5‑FU* 5‑fluorouracil, *OX* oxaliplatin

Of the 128 patients, 47.9% (58 of 121) subsequently received adjuvant chemotherapy, consisting of fluoropyrimidine (5-FU/leucovorin or capecitabine) in all patients except one. Sixty-four percent (37 of 58 patients) of those receiving adjuvant chemotherapy additionally received oxaliplatin. The proportion of patients receiving adjuvant chemotherapy did not differ significantly between BD-0 and BD-1 groups (*P* = 0.35; Fig. 3).

### Tumor budding and response

None (*n* = 20) or mild (*n* = 27) tumor budding (BD-0) was identified in the specimens of 47 (37%) patients and moderate (*n* = 52) or severe (*n* = 29) tumor budding (BD-1) in 81 patients (63%). There were 43 patients (33.6%) with no downstaging effect (T-level or nodal downstaging). Positive tumor budding (BD-1) was associated with significantly reduced T‑level downstaging (*P* < 0.001) and tumor regression as assessed by the four-tier AJCC/CAP tumor regression grading system (*P* < 0.001) [50]. With regards to tumor regression, a complete response with no residual viable tumor cells was achieved in 16 patients (13%) and a moderate response in 39 patients (30%), with only a single or small groups of cancer cells remaining. Nevertheless, over half of the patients had either minimal (42%, 54 of 128) or poor response (15%, 19 of 128).

Patients with BD-1 had a statistically significant association with AJCC/CAP tumor regression grade (*P* < 0.001), ypT (*P* < 0.001), ypN (*P* = 0.01), nodal involvement (*P* = 0.02), lymphatic invasion (*P* = 0.003), and ypUICC stage (*P* < 0.001; Table 2).

### Follow-up and events

All patients were followed at least for 5 years or to the date of death or loss to follow-up. A complete follow-up was achieved in 83% of patients as assessed by the completeness of follow-up index C as described by Clark et al. [49]. During the median follow-up time of 7 years (range 2.9–146.7 months), twenty patients (15.6%; BD-0: *n* = 4 and BD-1: *n* = 16) died. Of these, six patients (30%; BD-0: *n* = 1 and BD-1: *n* = 5) died within the first year. Overall tumor recurrence was diagnosed in 15 of the 128 patients (11.7%; BD-0: *n* = 2 and BD-1: *n* = 13). Locoregional recurrence occurred in six (4.7%), distant metastasis in twelve (9.4%), and combined locoregional and distant recurrence in three patients (2.3%).

### Time to event analyses

**Fig. 4:**
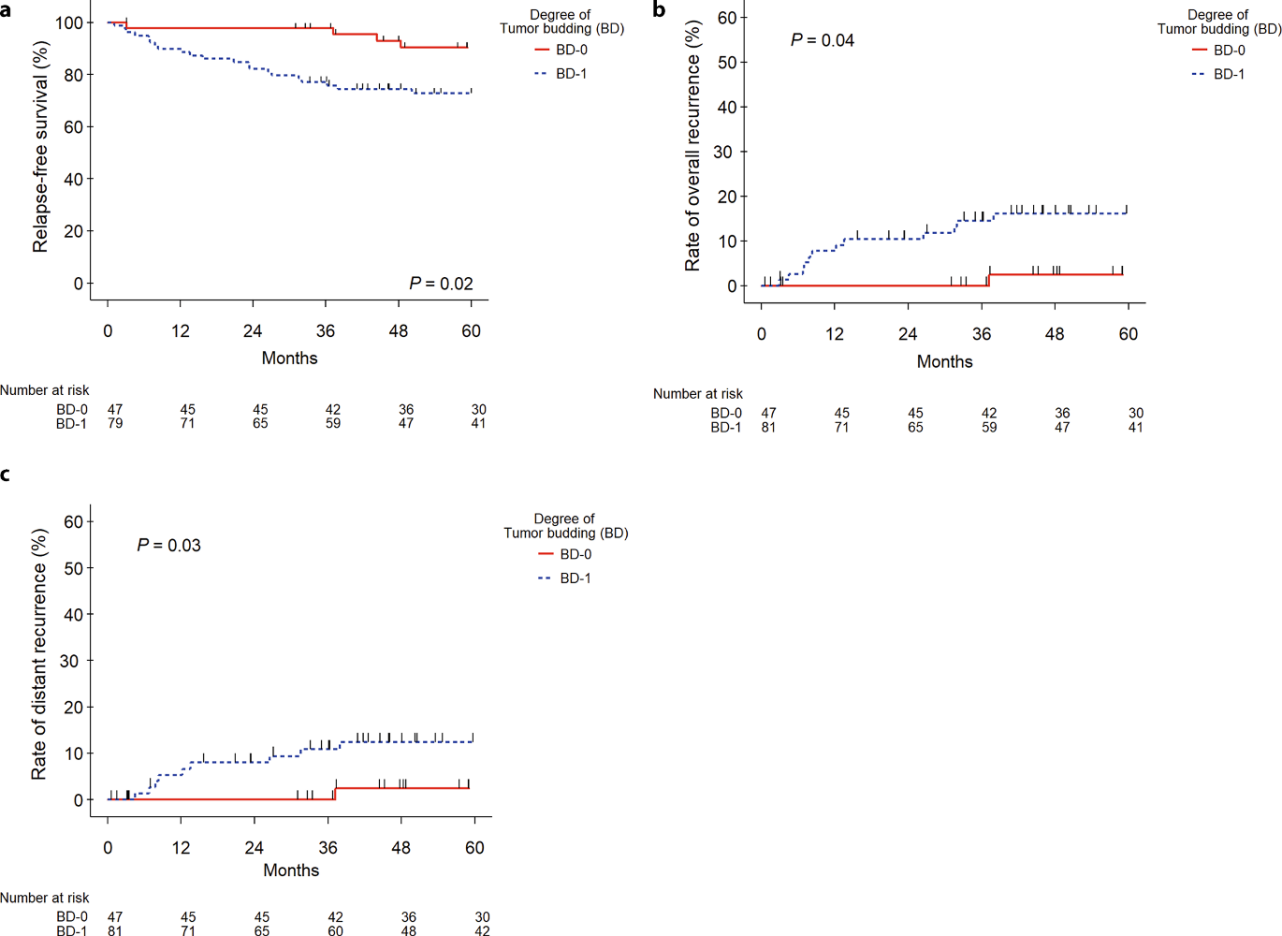
Rates of 5‑year relapse-free survival (**a**), overall recurrence (**b**), and distant recurrence (**c**) of 128 patients diagnosed with locally advanced non-metastasized rectal cancer who underwent preoperative chemoradiotherapy combined with total mesorectal excision

BD-1 patients had a considerably poorer 5‑year RFS, OR, and DR rates compared to BD-0 patients (RFS: 71% vs. 90%; *P* = 0.02; OR: 16% vs. 2%; *P* = 0.04; and DR: 12% vs. 2%; *P* = 0.03, respectively; Fig. 4a–c, Table 3). In univariate analyses, several variables, i.e., tumor budding, ASA classification, secondary malignancies, age, ypT, and circumferential resection margin, were significantly associated (*P* < 0.05) with RFS (Table 4). In multivariate analysis, only tumor budding, ASA classification, and circumferential resection margin were independently prognostic for RFS (Table 5). The results from this study failed to demonstrate a statistically significant association between BD-1 and OS and LR (*P* *=* 0.09 and *P* *=* 0.13, respectively).

**Table 3:** Five-year survival and recurrence estimates

			5-year estimates (95% CI)						
Parameter	Events	*n*	All grades^a^		BD-0 (*n* = 47)		BD-1 (*n* = 81)		*P*-value^b^
Overall survival	20	126	84	(76–89)	90	(76–96)	80	(69–87)	0.09
Relapse-free survival	27	126	78	(70–84)	90	(76–96)	71	(60–80)	0.02
Overall recurrence	15	126	11	(7–18)	2	(0–16)	16	(9–26)	0.04
Local recurrence	6	126	5	(2–11)	0	–	7	(3–17)	0.27
Distant recurrence	12	126	9	(5–16)	2	(0–16)	12	(7–23)	0.03

^a^Values shown are percentages and 95% confidence interval estimates (95% CI)

^b^Log-rank test, *BD* tumor budding

**Table 4:** *P*-values of univariate Kaplan–Meier analysis of patient, tumor, and treatment characteristics

Parameter	5-year RFS (%)	*P*-value^a^
*BD*		**0.015**
None/minimal (BD-0)	90	
Moderate/severe (BD-1)	71	
*Age, years*		**0.03**
≤64	86	
>64	68	
*Gender*		0.23
Female	85	
Male	74	
*ASA classification*		**0.002**
ASA 1 and 2	84	
ASA 3	54	
*BMI (kg/m* ^*2*^ *)*	–	0.64
*Surgical procedure*		**0.05**
APE	67	
LAR	82	
*Laparoscopic*		**0.08**
No	73	
Yes	88	
*Adjuvant chemotherapy*		0.22
No	73	
Yes	83	
*Time CRT to surgery (weeks)*	–	0.97
*Other malignancies* ^*b*^		**0.01**
No	81	
Yes	61	
*ypUICC*		**0.01**
0/I	86	
II	67	
III	78	
*ypT*		**0.02**
0/1/2	87	
3/4	70	
*ypN−/+*		0.84
Negative	78	
Positive	78	
*Angiolymphatic invasion*		0.97
No	77	
Yes	76	
*Grading*		0.80
1	100	
2	78	
3	77	
*CRM*		**<0.001**
>1 mm	80	
≤1 mm	33	

*ASA* American Society of Anesthesiologists, *BMI* body mass index, *BD* tumor budding, *CRT* chemoradiotherapy, *CRM* circumferential resection margin, *RFS* relapse-free survival, *UICC* Union for International Cancer Control

^a^Log-rank test. Parameters eligible for multivariate analysis (*p*-value ≤0.10) are indicated in bold

^b^Diagnosis of another malignant disease before (*n* = 6), at (*n* = 6), and after (*n* = 6) surgery for rectal cancer

**Table 5:** Multivariate analysis of factors influencing relapse free survival

		95% CI for HR		
	HR	Lower	Upper	*P*-value
*Tumor budding*				0.02
BD-0 (none/mild)	1	–	–	–
BD-1 (moderate/severe)	3.44	1.23	9.63	–
*ASA classification*				0.003
ASA 1 and 2	1	–	–	–
ASA 3	3.23	1.48	7.08	–
*CRM*				0.02
>1 mm	1	–	–	–
≤1 mm	3.09	1.21	7.89	–

*ASA* American Society of Anesthesiologists, *BD* tumor budding, *CI* confidence interval, *CRM* circumferential resection margin, *HR* hazard ratio, *RFS* relapse free survival

## Discussion

Tumor budding has been described most extensively in early and advanced colorectal cancer, implicating several scenarios in which this morphological feature might influence clinical decision making [33]. First, tumor budding could serve as a predictor of lymph node metastases in malignant polyps, which would suggest the need for resection. Second, budding may be indicative of tumor progression in stage II colorectal cancer to stratify for adjuvant therapy. Finally, tumor budding could be relevant as a predictor of tumor response to neoadjuvant therapy in pretreatment biopsies of locally advanced rectal cancer specimens [45, 46].

The first step in the development of a tumor bud seems to be its detachment from the main tumor body by loss of the adhesion molecule E‑cadherin [51]. Overall, EMT is implicated in the underlying molecular mechanism of budding, which includes loss of E‑cadherin in addition to the expression of fibronectin in the cytoplasm. These changes are suggestive of a more mesenchymal phenotype and also suggest a more aggressive tumor bud [51, 52]. Dysregulation of cell stemness phenotype (e.g., β‑catenin and CD133), cell–cell interaction (e.g., CD44 and E‑cadherin), and cell–matrix interaction (e.g., matrix metalloproteinase) as well as of inflammation (e.g., cytotoxic T cells) are essentially involved in tumor budding [53]. Analysis of these markers in pretreatment (and rarely post-pretreatment) specimens of colorectal cancer suggests that they could be linked to radio-chemoresistance via selective EMT properties [24, 54, 55]. Based on observations of existing precursors, it has been hypothesized that the EMT process is initiated through alterations induced by radiotherapy [24, 56]. It remains unclear how the underlying molecular mechanisms observed in EMT—and therefore in tumor budding—could either influence the effectiveness of chemoradiotherapy or, in turn, be influenced by chemoradiotherapy [57]. This was not, however, the purpose of the present study.

The aim of this study was to assess the impact of tumor budding, analyzed in surgical specimens, on oncological outcome and its possible role as a stratification parameter for a modified neoadjuvant therapy in chemoradiation-insensitive non-metastasized locally advanced mid-to-low rectal cancer patients.

Our data demonstrate a significant correlation between tumor budding, tumor regression, and downstaging after nCRT. In this respect, the current findings are consistent with the existing scarce literature investigating the role of tumor budding in neoadjuvantly treated rectal cancer patients (Table 6; [24, 37–40]).

**Table 6:** Literature review of articles investigating tumor budding in neoadjuvantly treated rectal cancer patients^a^

	Huebner et al. [37]	Du et al. [39]	Bhangu et al. [24]	Sannier et al. [38]	Present study
Design	RS	RS	RS	RS	RS
Publication year	2012	2012	2013	2014	2018
Institute	Mayo Clinic, Rochester, USA	Beijing Cancer Hospital China	Imperial College, London, UK	Beaujon Hospital, Clichy, France	PMU, Salzburg, Austria
Period	1996–2006	2001–2005	2009–2011	2005–2010	2003–2012
Intent	Curative	Curative	Curative	Curative	Curative
Patients (*n*)	237	96	69	113	128
Age (years)	Mean 60	Median 57	14 of 54 > 65	Median 59	Mean 64
Type of tumor	Primary	Primary	Recurrent: *n* = 15 Primary: *n* = 54	Primary	Primary
Tumor location (from anal verge)	≤12 cm	≤1 cm	–	≤12 cm	≤12 cm
Tumor stage	I, II, III	II, III	Locally advanced	I–IV	II, III
Neoadjuvant therapy	CRT	RT	CRT	CRT	CRT
Radiation scheme	Not mentioned	3000 cGy in 10 fractions in 2 weeks (36 Gy)	Fractionated, maximum dose of 54 Gy	45–50 Gy over 5–6 weeks	45–50 Gy over 5–6 weeks
Concurrent chemotherapy	5-FU	–	5-FU or capecitabine	5-FU	5-FU, capecitabine, Oxaliplatin
Interval to surgery (weeks)	6–8	2–3	≤6	6–9	3–9
Postoperative chemotherapy	233 (98.3%)	All patients	Not found	If ypN+ staged or distant metastases	47.9% (58 of 121)
Tumor budding present	24 (10.1%)	36 (37.5%)	25 of 45 (55.5%)	25 (22.1%)	81 (63.2%)
Median follow-up (years)	3.5	5.9	–	2.9	7
Associated with	CSS, RFS	DFS	OS, CSS	LR	DR, OR, RFS
Local recurrence	6 (2.5%)	–	–	5 (4.6%)	6 (4.7%)
Distant recurrence	43 (18.1%)	–	–	30 (27.8%)	12 (9.4%)

*CRT* chemoradiotherapy, *CSS* cancer-specific survival, *DFS* disease-free survival, *DR* distant recurrence, *Gy* Gray, *LR* local recurrence, *OR* overall recurrence, *OS* overall survival, *RFS* relapse-free survival, *RS* retrospective, *RT* radiotherapy.

^a^Not indicated Jessberger et al. [40]

In our cohort, we observed a T-level downstaging in 51.6% (65 of 126) of patients, together with an N‑level downstaging in 65.2% (43 of 66 clinically involved lymph nodes) of patients, accounting for an overall response rate (T-level or N‑level downstaging) of 64.3% (81 of 126). This is in accordance with the literature, where downstaging rates of 28–62% are reported [58, 59]. Patients with BD-0 experienced a T-level response rate of 82.6% (38 of 46) compared to those with BD-1 of 33.8% (27 of 80; *P* < 0.001).

Bhangu et al., who investigated the association between EMT (which is thought to be the underlying molecular mechanism behind tumor budding) and non-response in a cohort of 69 (primary: *n* = 54 and recurrence: *n* = 15) rectal cancer patients who were curatively treated with nCRT and surgery, reported a non-response rate of 65%. In the subgroup analysis of 54 primary cancers, all EMT biomarkers and tumor budding have been investigated in 45 patients and found to be positive in 24 of 31 non-responders (77.4%) compared to 1 of 14 responders (7.1%; *P* < 0.001), indicating a significant association with non-response [24]. Huebner et al., who investigated the impact of pathologic parameters in a cohort of 237 nCRT-treated rectal cancer patients, were the first to systematically study tumor budding in rectal cancer after neoadjuvant therapy. They found tumor budding to be a significant predictor of survival [37]. In contrast to our findings of a budding rate of 63%, they reported a budding rate of only 10%. This discrepancy may be explained by their definition of tumor budding, which was positive if any field (counted at 200 × magnification using a routine H&E staining) had 10 or more buds, a method described by Ueno et al. [60]. In our cohort, tumor budding was defined as positive (moderate or severe budding, BD-1) if one or more foci in an average of 10 HPF were observed [46, 47, 61, 62]. This method of defining tumor budding can be contrasted to that described by Sannier et al., who only recorded the absence or presence of tumor buds without any cut-off value to define tumor budding. They explained this by the ease of the applicability and the association of even low numbers of tumor buds with adverse effects on outcome in nCRT-treated patients [38]. Irrespective of the varying tumor budding rates, the abovementioned three studies—to the authors’ best knowledge—are the only existing studies that explicitly investigated the prognostic role of tumor budding in nCRT-treated rectal cancer patients (Table 6; [24, 37, 38]). Jessberger et al. demonstrated the prognostic value of tumor cell growth patterns (which included tumor budding) in predicting survival in post-CRT surgical specimens, but not in pretreatment biopsies [40]. Although this study group used a different technique to identify tumor budding, the alteration of cancer cells’ growth pattern along the invasion front by nCRT was demonstrated, indicating potential constraints in assessing posttreatment tumor budding [40].

Tumor budding is usually assessed at the tumor invasive front, as buds are most prominent here [33]. In contrast to colorectal cancer specimens without nCRT, pathological evaluation of rectal cancer after chemoradiation is more complex. It is important to consider that residual cancer cells in nCRT-treated rectal specimens are unequally distributed in the bowel wall [63], and an invasive front is not necessarily present to correctly score tumor budding in rectal cancer after nCRT [38]. In our cohort, specimens with clear radiogenic regression (AJCC/CAP grade 0, “complete response”) exhibited destruction of the tumor glandular component with subsequent fibrosis resulting in disruption of the tumor tissue, which made budding assessment more challenging, a topic first investigated and described by Du et al. [39]. Nevertheless, in the remaining regression grades (AJCC/CAP 1, 2, and 3; “moderate,” “mild,” and “poor” responses, respectively), the assessment of tumor budding was possible as the tumor invasive front could be clearly identified according to the 10 HPF method [46, 47, 62]. Du et al. in their study evaluated the morphology and prognostic value of tumor budding in 96 rectal cancer patients after radiotherapy alone and consecutive curative resection. Tumor budding in irradiated specimens was found to be an independent factor, among others, affecting long-term disease-free survival. They further demonstrated an excellent concordance of tumor budding assessed on H&E and immunohistochemical stained slides of irradiated rectal cancer specimens, indicating the feasibility of tumor budding assessment on H&E stained slides of irradiated specimens [39].

A drawback of tumor budding assessment and reporting is the limited reproducibility and the lack of standardization with various existing techniques for assessing and classifying tumor budding, as excellently reviewed recently [64–66]. Sources of variability in the assessment of tumor budding include the optimal location for assessment (tumor front vs. within the tumor), visualization, and staining (H&E staining vs. immunohistochemistry) of the budding cells, as well as the method of scoring (qualitative vs. quantitative) [67].

Intratumoral budding was demonstrated to be an independent prognostic factor and strongly correlated with peritumoral budding in a cohort of 511 colorectal cancer patients, supporting the future relevance of intratumoral budding, especially in preoperative rectal cancer specimens [68]. This was confirmed by a retrospective study conducted by Rogers et al., who assessed the intratumoral budding in pretreatment rectal cancer biopsies with a budding rate of 20% (18 of 89) and confirmed tumor budding to be a predictive factor for a poor pathological response to nCRT (higher ypT stage, lymph node involvement, lymphovascular invasion, and residual poorly differentiated tumors) and long-term outcome [29].

Although the evaluation of tumor budding with immunohistochemistry has improved detection rates and interobserver agreements compared to H&E staining, the International Tumor Budding Consensus Conference recommends tumor budding assessment using H&E [45]. Indeed, in some previous meta-analyses, the prognostic impact of tumor budding assessed using H&E did not differ significantly compared to immunohistochemistry [28, 33, 46, 64, 66].

Despite the existence of the aforementioned variability in the definition of how many cells constitute a tumor bud and how many buds constitute positive budding, together with the considerable interobserver variability in its reporting, most studies definitively demonstrate tumor budding as a strong negative prognostic marker in colorectal cancer [64]. Additionally, the results of the International Tumor Budding Consensus Conference (April 27–29, 2016, Bern/Switzerland) provide optimism that an agreement on an international, evidence-based standardized scoring system for tumor budding in colorectal cancer is on the horizon [45].

There are several limitations to our study. First and foremost are the limitations inherent to retrospective analyses. The number of events in relapse-free survival may be a limitation in the interpretation of the multivariate analysis. In our cohort study, the completeness of follow-up in the two groups (BD-0 and BD-1) was 88% and 81%, indicating data incompleteness and a possible bias in the analysis.

To date, the assumption that a tumor budding status following nCRT reflects its dimension before any treatment is based on one study only and should be validated in larger cohorts at other institutions [29]. Nonetheless, in the row of the few reports dealing with the role of tumor budding in neoadjuvantly treated rectal cancer patients, the present analysis provides robust data with the longest follow-up time (median: 7 years, with each patient followed at least for 5 years) and the most current treatment era (until the end of 2012) and thus treatment regimen.

## Conclusion

Our data confirm the predictive value of tumor budding persistence after neoadjuvant chemoradiotherapy and, hence, putatively for the response towards nCRT in locally advanced rectal cancer patients. In addition to the established predictive parameters (TNM classification and tumor regression grading), tumor budding is accessible in pretreatment biopsy specimens and could therefore, at least hypothetically, serve as a predictor of non-response to nCRT, also providing a possibility to stratify putative non-responders into an individualized nCRT regimen.

Further efforts are needed to gather scientific evidence to validate the promising role of tumor budding and to gain a greater understanding of the underlying molecular mechanisms behind EMT and tumor budding. This information will help to improve the multidisciplinary treatment of locally advanced rectal cancer patients.
